# Self-Medication Practice and Associated Factors Among Health Science Students in Central Tanzania

**DOI:** 10.24248/eahrj.v8i3.810

**Published:** 2025-01-30

**Authors:** Elihuruma E Stephano, Osward S Lyimo, Victoria M Godfrey, Silas G Shemdoe, Masanyiwa E James, Rajabu M Kingo, Julius E. Ntwenya

**Affiliations:** a Department of Clinical Nursing, School of Nursing and Public Health, The University of Dodoma, Dodoma, Tanzania; b Department of Nursing Education and Management, School of Nursing and Public Health, The University of Dodoma, Dodoma, Tanzania; c Directorate of Nursing Services, Dodoma Regional Referral Hospital, Dodoma, Tanzania; d Department of Pharmacy, School of Medicine and Dentistry, The University of Dodoma, Dodoma, Tanzania; e Department of Physiology and Biochemistry, School of Medicine and Dentistry, The University of Dodoma, Dodoma, Tanzania; f Department of Public Health and Community Nursing, School of Nursing and Public Health, The University of Dodoma, Dodoma, Tanzania.

## Abstract

**Background:**

Self-medication practice is highly prevalent worldwide despite the scaled-up campaigns to refrain from using drugs without a prescription. Inadequate knowledge has been associated with the increasing practice of self-medication among students. The study aimed to determine the prevalence of self-medication and explore factors associated factors with the practice among health science students.

**Methods:**

A cross-sectional study was conducted in Dodoma, where 255 students were interviewed using a self-administered adapted questionnaire. Univariate and multivariate analysis were performed using SPSS version 25.

**Results:**

Self-medication prevalence was 69%. Paracetamol (52.1%) and Ibuprofen (29.4%) were primarily used for relief of headache (76.7%) and menstrual pain (18.2%). Among the studied students, 59.6% had inadequate knowledge, while 60.8% had a positive attitude towards self-medication. Mode of hospital payment (cash) (AOR=3.75; 95% CI: 1.868–6.825 *p<.001*) and household income (<10,000TSh) (AOR=2.868; 95% CI: 1.355–6.071 *p=.006*) were significantly associated with self-medication practice.

**Conclusion:**

Self-medication practice among health science students is prevalent. Inadequate knowledge and low socioeconomic status play a significant role in self-medication practice. Increasing students’ access to inexpensive healthcare options and counselling services may help in reducing self-medication practices.

## BACKGROUND

The World Health Organization (WHO) defines self-medication as the act of treating self-diagnosed ailments without professional supervision using non-prescribed drugs. Self-medication is a concerning global public health issue with a prevalence ranging from 11.7 to 92% across the world.^1^ Individuals often rely on their intuition or advice from friends and family, using leftover medications from home or purchasing them from pharmacies. Some may use old prescriptions to address current health problems.^2^ Numerous studies conducted in various countries have investigated self-medication practices among different population groups.^2–4^ These studies have highlighted issues such as therapeutic failure, increased patient costs, a rise in unnecessary side effects, and the development of antibiotic resistance, which makes illnesses more difficult to treat.^1,5,6^

Drug consumption through self-medication is very high in low and middle-income countries.^6^ The majority resort to self-medication for health needs due to limited public health systems and policies, the unregulated sale of over-the-counter (OTC) drugs, the high prevalence of infectious diseases, limited access to appropriate medications, and inadequate diagnostic tools.^7^ Lack of awareness of the adverse effects of drugs has been reported to influence self-medication practice.^2^ Despite the well-documented global prevalence and causes of self-medication practices, specific data on health science students in Central Tanzania remains scarce. The prevalence of self-medication with antibiotics was reported to be 23.6% and 23.4% in rural and urban Dodoma, respectively.^7^ Evidence indicates that the prevalence of self-medication is higher in non-medical students (46.2%) than in medical students (35.1%).^8^ However, this study focused only on antibiotic use, which requires understanding general practice.^8^ Health science students are expected to acquire medical knowledge and may have distinct attitudes and behaviors toward self-medication compared to the general population.^3^ It is crucial to understand the practice of health sciences students as they represent the future healthcare workforce, and their habits and knowledge will significantly impact public health. This study was conducted to determine the prevalence and factors associated with self-medication among health science students in Central Tanzania.

## METHODS

### Study Design, Setting and Population

A cross-sectional study was conducted in the Dodoma city council in the Dodoma region of Central Tanzania, targeting health science students enrolled in diploma-level training programs. The Dodoma Region, being a central hub for education and healthcare training in Tanzania, provided a diverse and representative sample of students. Dodoma City Council has four health and allied institutes and two universities that are offering health and allied sciences programs. All health institutions were involved in this study. Based on the institutional classification, there are two universities (the University of Dodoma [UDOM] School of Medicine and School of Nursing and Public Health, and St. John’s University of Tanzania [SJUT]), two colleges (Decca College of Health and Allied Sciences [DECOHAS] and City College of Health and Allied Sciences [CICOHAS]), and two institutes (Dodoma Institute of Health and Allied Sciences [DIHAS], and Benjamin Mkapa Institute of Health and Allied Sciences [BMIHAS]). Students in their first, second, and third years who were enrolled in medical laboratory, nursing, midwifery, pharmacy, environmental health, and community health programs were included in this study.

### Sampling Technique

Participants were randomly chosen from six Dodoma colleges offering health science programs. The study employed a stratified random sampling technique to ensure a representative sample of health science students in the Dodoma Region. The stratification process utilized two primary strata, which are the educational institution and program level. The sampling population consisted of students enrolled in health science programs across several educational institutions in the city.

The sample size determination followed the principle of proportional representation based on institutional enrolment. Following the principle of proportional allocation in stratified sampling, where sample sizes are determined relative to the population size of each stratum, the 255 participants were distributed as follows: UDOM with 61 participants (23.9%), closely followed by SJUT with 59 participants (23.1%). DECOHAS provided 42 participants (16.5%), while CICOHAS contributed 33 participants (12.9%). DIHAS contributed 39 participants (15.3%), and BMIHAS provided 21 participants (8.3%). This study ascertained the number of health science students from each selected college and their contacts’ names and addresses with the assistance of Class Representatives. A study participant was chosen at random to participate in an interview. The identified potential respondents were asked to accept participation in the study; if they declined, a different person was randomly selected to take their place. The students who participated in the final interview were chosen using a formal random sampling process.

### Sample Size

**Figure 1.**
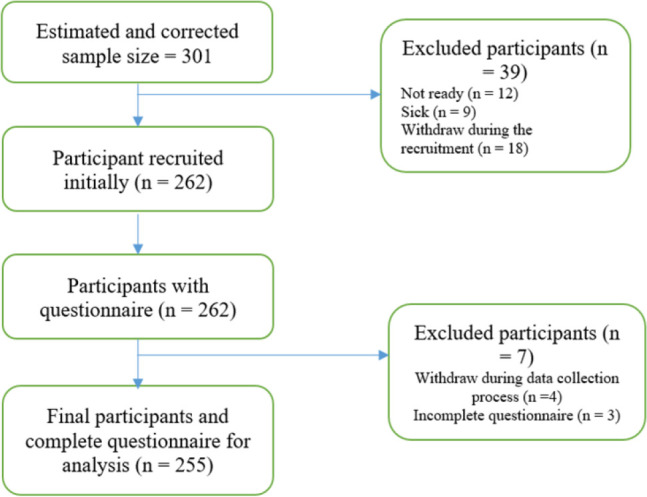
The Flow Pattern of Sample Size Estimation and Recruitment

A single population proportion formula (*n*_*i*_ = *z*^2^*p* (1−*p*)/*e*^2^) was used to determine the sample size for this investigation. Due to the lack of accurate numbers from the previous research report, we calculated that the proportion of self-medication (*p*) = 0.5 (50%) of the general population engages in self-medication, with the assumptions (z) = 1.96 for 95% confidence interval, and a required margin of errors (e) 0.05 (5%). This resulted in an initial sample size of 384; however, the total number of students (N) was relatively small in the study area, estimated to be 1131,^9^ so a correction factor was introduced as n_c_ = (n_i_*N)/(N + n_i_)), which gave a sample size of 287. After adding 5% for non-response, n_3_, the final sample size was 301. We could sign up 255 people with full participation and eligibility criteria, yielding an 84.7% response rate. The flow pattern of sample size estimation and final recruitment are highlighted in Figure 1.

The sample size was determined based on the total number of students in these programs, using appropriate statistical methods to ensure sufficient power to detect significant associations between self-medication practices and the various factors being studied.

### Data Collection Procedure

Data was collected between July and September 2022 using self-administered questionnaire. Based on the information obtained from class representatives in each participating institution, researchers employed a stratified sampling technique to get the required sample size in each visited institution. Informed consent was sought prior to administration of the questionnaire. Consented students were instructed to complete the self-administered structured questionnaire. Students who expressed a desire not to participate in the study or could not complete the questionnaire throughout the data collection process were considered non-respondents. Statistical Package for Social Science (SPSS), version 25, was used to handle and analyze the actively gathered data.

### Data Collection Tool

This study employed a standardized, self-administered questionnaire with four elements to evaluate self-medication usage and its contributing factors. The four sections comprised eight socio-demographic and socioeconomic questions, ten questions about self-medication and its effects, eight about attitudes, and three about practice. The questionnaire was prepared after carefully reviewing the literature, and most of the questions were taken from related past studies based on the objectives. The questionnaire was examined for face and construct validity by three experts. The questionnaire is initially composed in English, translated into the regional language (Swahili), and then returned to English to ensure uniformity. A pre-test was taken by 35 participants three weeks before the data collection who were not included in the final analysis.

### Operational Definition

Self-medication practice was defined as any use of drugs for treating self-diagnosed diseases without a prescription from a health care worker with a government-issued license to write prescriptions.

### Variables and variables measurement

The socio-demographic and socioeconomic factors (age, gender, income, health insurance, year of study, married status, religion, and prior knowledge), attitude toward self-medication, and knowledge about self-medication practices and consequences are the independent variables of this study. The use of self-medication is the dependent variable(s).

The questionnaire used to measure the variables was taken from an Ethiopian study that evaluated knowledge, attitude, and self-medication behaviour, and it was adapted to fit the study’s design. Respondents had to select either “Yes” or “No” from the options given in knowledge-based questions. The respondents were divided into two groups based on their mean knowledge scores on self-medication and its effects (inadequate and adequate knowledge). Respondents who selected “Yes” scored 1 for having successfully answered the question, while those who selected “No” scored 0 for not responding correctly. Respondents were categorized as having appropriate knowledge if their scores were equal to or higher than the mean of the correctly answered questions; otherwise, they were classified as having insufficient knowledge.

In the attitude measurement, each of the eight statements was supported using self-medication. On a Likert scale, the respondent might select one of five options (5 = strongly agree, 4 = agree, 3 = not sure/neutral, 2 = disagree, and 1 = severely disagree). An overall higher mean score denotes a good attitude toward self-medication practice. The total score ranged from 8 to 40.

### Data Analysis Procedure

The completed data were reviewed, organized, coded, and loaded into the SPSS version 25 software for analysis. The socio-demographic factors, degree of knowledge, attitude, and practice were described using descriptive statistics. Cross-tabulation was used to disperse the participant characteristics in the self-medication practice, and a Chi-square test was produced. Binary logistic regression, including univariate and multivariate variables, was conducted to identify factors linked to self-medication. The descriptive results were presented using text narration and tabulation. The multivariate logistic regression analysis determined which variables were statistically significant for the dependent variable. The adjusted odds ratio (AOR) and 95% confidence interval (CI) demonstrated the strength of the association.

### Ethics approval and consent to participate

Ethical approval was obtained from the Institution Review Board of the University of Dodoma (Reference Number: MA.84/261/01/62/332). The local government issued a letter of approval for participant data collection. The study’s informed consent procedures, which included collecting written and oral consent, effectively protected the participant’s rights. Participants were advised that participation was optional, that no payment would be made, that there was no experiment involving human tissue samples, and no adverse effects while enrolled in the study. The respondents who agreed to participate in the study were guaranteed the confidentiality of the information.

## RESULTS

### Students’ Socio-demographics

Table 1 shows the socio-demographics of 255 participants. Most of the participants were single (81.2%) and within the age group of 22 to 25 years. Those who paid for health services comprised more than half of the study participants (58.8%), and 166 (65.1%) were off-campus students.

**Table 1:** Socio-Demographic Characteristics of Students (N=255)

Variable	N	%
Age group		
18–21	19	7.5
22–25	161	63.1
>26	75	29.4
Gender		
Male	79	31.0
Female	176	69.0
Marital status		
Married	48	18.8
Single	207	81.2
Year of study		
First-year	69	27.0
Second-year	94	36.9
Third-year	92	36.1
Home daily income		
Below 10,000TSh (4 USD)	117	45.9
11,000–20,000TSh (5–9 USD)	84	32.9
Above 21,000TSh (10 USD)	54	21.2
Mode of hospital payment		
Health insurance	105	41.2
Cash	150	58.8
Institution/College/University		
UDOM	61	23.9
SJUT	59	23.1
DECOHAS	42	16.5
CICOHAS	3 3	12.9
DIHAS	39	15.3
BMIHAS	21	8.3
Accommodation		
Off-campus hostel	166	65.1
Home premises	89	34.9
Course of study		
Medical science	100	39.2
Applied science	155	60.8
OTC = Over the Counter		

### Knowledge of Self-medication and Its Implications

The mean score for knowledge was 5.82±2.16, with the score ranging from 0 to 10. Generally, students had inadequate knowledge, as 59.6% had scored below the mean score. While the majority knew that self-medication causes harmful side effects (81.2%) and new illnesses (73.3%), only 29.4% knew that they were supposed to immediately stop using the drug and report to health care providers if side effects were observed. Few students (22.0%) knew that not all diseases can be treated by over-the-counter drugs (Table 2).

**Table 2:** Knowledge of Self-Medication Among Students (N=255)

Knowledge items	N	%
Ql. OTC drugs are not safe and effective	109	42.7
Q2. Self-medication can cause new illness	187	73.3
Q3. Not all diseases can be treated by OTC drugs	56	22.0
Q4. OTC drugs could not be used after expired date	96	37.6
Q5. Not safe to take OTC drugs with other prescribed drugs	91	35.7
Q6. Self-medication can cause addiction	207	81.2
Q7. Self-medication delays to seek medical care	170	66.7
Q8. Self-medication cause drug resistance	159	62.4
Q9. During pregnancy OTC should be used cautiously	95	37.3
Q10. Stop using drugs immediately and report If noticing side effects	75	29.4
Overall knowledge level Inadequate knowledge	152	59.6

### Students’ Attitude Towards Self-medication Practice

The mean score for attitude was 26.0±4.91, ranging from the lowest, 8, to the highest, 40. Overall, 39.2% of the respondents had a negative attitude toward self-medication (scored below the mean score). Over one-third of students (38.4%) disagreed that it is appropriate to share medication with others.

### Prevalence of Self-medication Practice and Associated Factors.

The prevalence of reported self-medication for several medical conditions among students was 69% which translates to approximately 176 students. Within this group, the male proportion of self-medication was 66.5%, accounting for about 117 males, while the female proportion was 33.5%, resulting in approximately 59 females. The practice of self-medication significantly varied with the mode of healthcare payment and household socioeconomic status (Table 3).

**Table 3:** Cross Tabulation of Respondents’ Socio-demographics Self-medication Practice

Variable	Practice self-medication				*p-value*
	Yes		No		
	n	%	N	%	
Age group					0.318
Adolescent	15	78.9	4	21.1	
Young adult	106	65.8	55	34.2	
Late adult	55	73.3	20	26.7	
Gender					0.190
Male	59	74.7	20	25.3	
Female	117	66.5	59	33.5	
Marital status					0.153
Married	19	39.6	29	60.4	
Single	60	29.0	147	71.0	
Course of study					0.575
Medical science	67	67.0	33	33.0	
Applied science	109	70.3	46	29.7	
Year of study					0.854
First-year	49	71.0	20	29.0	
Second-year	63	67.0	31	33.0	
Third-year	64	69.6	28	30.4	
Accommodation					0.062
Off-campus Hostel	108	65.1	58	34.9	
Home premises	68	76.4	21	23.6	
Home daily income					0.001
10,000 and below	94	80.3	23	19.7	
11,000–20,000	51	60.7	33	39.3	
Above 21,000	31	50.0	23	50.0	
Mode of hospital payment					<0.001
Health insurance	55	52.4	50	47.6	
Cash	121	80.7	29	19.3	
(N=255)					

### Predictors of Self-medication Practice

During univariate logistic regression, gender, marital status, accommodation, mode of hospital payments, and home income met the set standard for multivariate regression (*p-value ≤.2*). However, in the multivariate logistic regression, only the mode of hospital payments and home income were significantly associated with self-medication practice (*p-value <.05*).

Students who paid hospital services in cash were more likely to practice self-medication than those who paid by health insurance (AOR: 3.57; 95% CI = 1.868 – 6.825). Students whose daily income was less than 10,000 Tshs (AOR: 2.868; 95% CI = 1.355 – 6.071) were likelier to practice self-medication than students whose home income was 20,000 Tsh or above (Table 4).

**Table 4.** Predictors of Self-medication Practice (N=255)

Variable	COR	95% CI		*p-value*	AOR	95% CI		*p-value*
		Lower	Upper			Lower	Upper	
Gender								
Male	Ref							
Female	0.672	0.37	1.22	0.191	0.885	0.457	1.714	0.716
Marital status								
Married	Ref							
Single	1.605	0.836	3.08	0.155	0.925	0.431	1.986	0.842
Accommodation								
Off-campus Hostel	Ref							
Home premises	1.739	0.97	3.119	0.063	1.736	0.918	3.282	0.089
Mode of hospital payment								
Health insurance	Ref							
Cash	3.793	2.172	6.624	<0.001	3.57	1.868	6.825	<0.001
Home daily income								
Above 20,000	Ref							
10,000–20,000	1.147	0.572	2.297	0.699	1.429	0.671	3.046	0.355
Below 10,000	3.032	1.496	6.145	0.002	2.868	1.355	6.071	0.006

## DISCUSSION

The practice of self-medication is generally widespread despite the fact awareness of possible drawbacks is high among the studied students. Other previously conducted studies in Dar es Salaam,^10^ Mwanza,^11^ College Station, Texas, US Ethiopia,^12^ and Eritrea^13^ have also reported high rates of self-medication practice. However, a low prevalence of OTC drug use was reported in Nigeria,^14^ Iraq,^15^ and Portugal.^4^ Differences in socioeconomic status, sample size and sampling procedure used, specific contextual environment, types of drugs investigated, and law enforcement may account for the observed variances between different studies.

The most frequently reported self-medication drugs were Ibuprofen and paracetamol, which are readily available without prescription^16^ for relief of pain and fever. Thus, most participants could purchase medications based on their symptoms and signs. Similar findings have been reported in other parts of the world.^17,18,19,20^

In line with our findings, other studies have also reported that people have a limited understanding of self-medication and its effects.^21,22^ However, a study conducted in Ethiopia reported excellent knowledge of the safety and efficacy of OTC medication among medical university students, which was linked to their exposure to medical literature.^17^

The association between increased self-medication practice and low household income may be attributed to students’ inability to meet the high cost of healthcare services. The use of self-medication might be encouraged by financial constraints and limited access to healthcare facilities.^8,18,23,24^ Other reasons for the high prevalence of self-medication may include drug accessibility and lengthy waiting times at health facilities.^23^ The high burden of out-of-pocket payments may make it more difficult for low-income individuals to access healthcare services and opt for self-medication.^25,26,27^ Results suggest that the cash payment method may unintentionally promote self-medication among medical students and the general population by limiting access to healthcare services. Therefore, increasing access to affordable healthcare services and health insurance may help reduce self-medication practices.

Limitations in this study include using a cross-sectional study design, which does not allow the establishment of causality associations. Additionally, the reliance on self-reported data may introduce recall and social desirability biases, potentially affecting the accuracy of the findings. Despite these limitations, the study provides a crucial foundation for understanding self-medication practices among health science students in Central Tanzania.

## CONCLUSION

Despite awareness of the potential risks, such as adverse drug reactions and the development of antibiotic resistance, a high proportion of students continue to self-medicate, primarily with readily accessible drugs like Ibuprofen and paracetamol. The prevalent self-medication is largely explained by students’ inability to afford the costs of healthcare services.

## RECOMMENDATION

Implementation of comprehensive health insurance and increasing access to counselling and affordable healthcare services may help to reduce self-medication practice among students.
